# Intramuscular myxoma associated with an increased carbohydrate antigen 19.9 level in a woman: a case report

**DOI:** 10.1186/1752-1947-5-184

**Published:** 2011-05-14

**Authors:** Dimitrios Theodorou, Eleftheria S Kleidi, Georgia I Doulami, Panagiotis G Drimousis, Andreas Larentzakis, Kostas Toutouzas, Stylianos Katsaragakis

**Affiliations:** 1 First Department of Propedeutic Surgery, University of Athens, Athens Medical School, Hippocration Hospital, Athens, Greece

## Abstract

**Introduction:**

Intramuscular myxoma is a rare benign soft tissue tumor. The lack of specific symptoms and widely used laboratory tests makes the diagnosis quite difficult. We present a case of an Intramuscular myxoma associated with an increased carbohydrate antigen 19.9 level. To the best of our knowledge, there have not been any reported cases of an association of Intramuscular myxoma with tumor markers in the literature.

**Case presentation:**

A 45-year-old Caucasian woman presented to our department for resection of a mass in her left groin area, discovered incidentally on a triplex ultrasonography of her lower extremities. The diagnosis of Intramuscular myxoma was confirmed on histopathology after the complete surgical excision of the tumor. On laboratory examination, the serum level of carbohydrate antigen 19.9 was found to be elevated, but it returned to normal six months after resection of the mass.

**Conclusion:**

Carbohydrate antigen 19.9 is a tumor marker that increases in a variety of malignant and benign conditions. After the exclusion of all other possible reasons for carbohydrate antigen 19.9 elevation, we assumed a possible connection of carbohydrate antigen 19.9 elevation and Intramuscular myxoma, an issue that requires needs further investigation.

## Introduction

Intramuscular myxoma (IM) is a rare benign soft tissue tumor that presents as a slowly growing, deeply seated mass confined to the skeletal muscle. IM has an incidence of 0.1 to 0.3 per 100,000 [1]. According to the World Health Organization, IM is classified as a tumor of uncertain differentiation [2]. The symptoms, if any, are usually vague. The only widely available diagnostic tests are imaging studies, such as ultrasonography, computed tomography (CT), and magnetic resonance imaging (MRI), which reveal a mass but cannot differentiate. The definite diagnosis of IM can only be made after its surgical excision, which is also agreed to be the treatment of choice [3].

We present the case of a 45-year-old Caucasian woman with an IM in her left groin area. It was diagnosed on histopathology after its complete excision. Pre-operative screening revealed an elevated carbohydrate antigen (CA) 19.9 level, which returned to normal six months after the surgical excision. To the best of our knowledge, an association of CA 19.9 with the diagnosis of an IM has not previously been considered. This hypothesis is presented after the exclusion of all other possible causes along with a brief review of the literature.

## Case presentation

A 45-year-old Caucasian woman was admitted to our surgical department for treatment of a mass in her left groin area. From her past medical history, our patient was on treatment with levothyroxine after thyroidectomy for multi-nodular goiter and with amlodipine and valsartan for hypertension. She did not smoke cigarettes and did not report any history of trauma in the area.

The mass was discovered incidentally on a lower extremity triplex ultrasonography one month before her admission. Our patient was complaining of aching, soreness and heaviness of her lower extremities for two months and was advised to have her lower extremity venous system evaluated. On her right lower extremity, the triplex ultrasonography revealed insufficiency of the saphenofemoral junction and insufficient valves of the great saphenous vein. On her left lower extremity, the study was difficult to perform because of a mass in the groin area. It was a solid hypoechoic mass of heterogeneous texture, 50×55 mm in size, lying 11 mm under the skin surface and with minimal blood flow. It appeared to be in proximity with the femoral vessels but without compressing them, and there was no local lymph node enlargement.

On physical examination, a painless, fixed, solid mass was palpated in her left groin area. Both lower limbs were symmetrical with normal motility.

**Figure 1:**
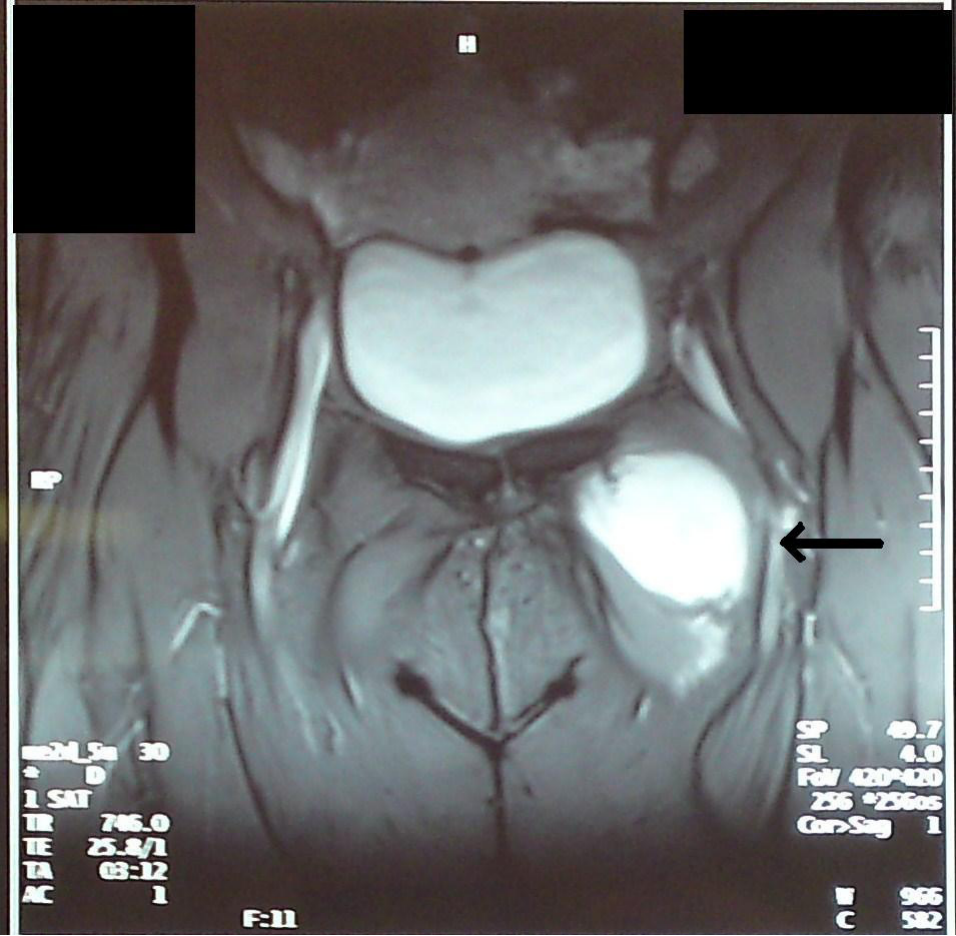
**Coronal T2-weighted MRI of left groin Intramuscular myxoma**.

**Figure 2:**
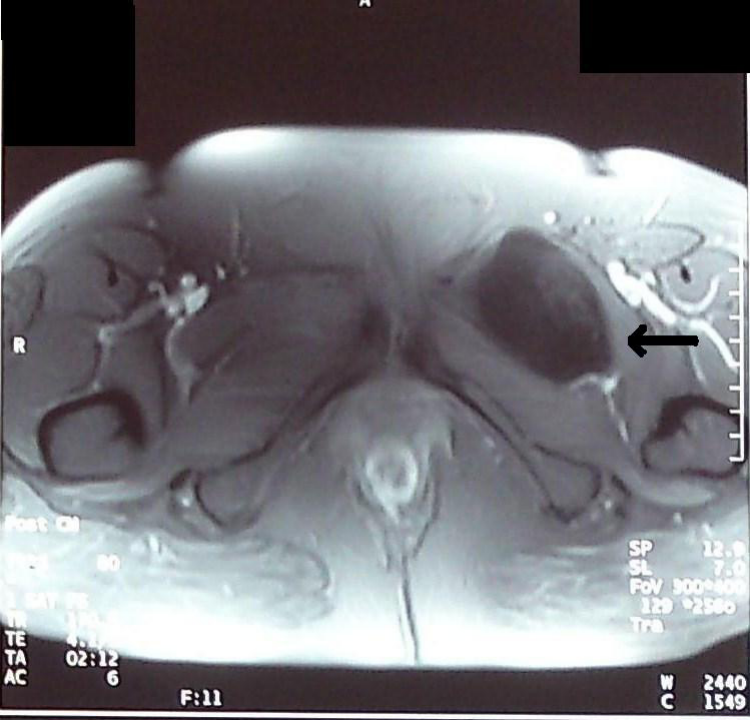
**Axial T1-weighted MRI of left groin Intramuscular myxoma**.

Our patient was subsequently submitted for an MRI of the area. It revealed a mass lying in a space defined anteriorly from her pectineus muscle, posteriorly from her abductor muscle, laterally from her obturator muscle and medially from her innominate bone. The mass had a heterogeneous low signal intensity on T1-weighted images and heterogeneous high signal intensity with inner areas of low signal intensity on T2-weighted images. It was lobulate with dimensions 78×59×45 mm and relatively well-defined margins. No enhancement was marked after the intravenous administration of paramagnetic substance (Figures 1 and 2). Additional imaging studies (upper and lower abdominal ultrasonography, chest radiography) did not reveal any other pathology.

On laboratory examination, a full blood count, basic metabolic panel, liver and kidney function tests, electrolytes and amylase, and coagulation profile were normal. The thyroid function tests showed euthyroidism. Cancer- and tissue-specific markers (α-fetoprotein, carcinoembryonic antigen, CA 15.3, CA 19.9 and CA 125) were also tested. Of these, CA 19.9 was found elevated at 39.51 U/mL (reference range, 0-35 U/mL). To exclude possible laboratory error, the elevated value of CA 19.9 was repeated twice.

Our patient underwent a surgical excision of the mass. A longitudinal incision over the femoral vessels was performed, and a mass measuring approximately 6 cm was identified. It was firmly attached to the adjacent structures; however, it was dissected without ligating any large blood vessel. The mass was resected *en bloc *and sent for histopathology study. The incision was closed with interrupted sutures.

The post-operative period was uneventful, and our patient was discharged on the third post-operative day.

The macroscopical specimen examination revealed an oval-shaped lobulated mass 80× 55×50 mm in size with residual striated muscle. On histopathology, it was found to be an IM.

At the follow-up six months later, our patient did not have any complaints, and there was no clinical evidence of recurrence. Ultrasonography of her left groin area revealed insufficiency of the saphenofemoral junction and no mass recurrence. Upper and lower abdominal ultrasonography results were normal. All laboratory test results were normal, and CA 19.9 was reduced to 11.34 U/mL. During colonoscopy, the sigmoid colon was found to be edematous and spastic, and first-grade hemorrhoid disease was present.

## Discussion

IM is a rare entity. Virchow introduced the term *myxoma *in 1863 to describe a tumor that in histology resembles the umbilical cord [4]. The initial criteria for diagnosis of myxoma was established by Stout in 1948 when he stated that myxoma is a true mesenchymal neoplasm composed of undifferentiated stellate cells in a myxoid stroma [4]. It is still not clarified if IM is a benign soft tissue tumor or a reactive proliferation of hypersecretory fibroblasts [5].

The majority of IMs appear from the fourth to sixth decades of life, with a slight predominance in women (male:female ratio, 1:1.4) [4]. It usually arises from large skeletal muscles, so the commonest location is the lower extremities, particularly the thigh (51%) and the gluteal area (7%) [4,6]. IMs can measure up to 20 cm; however, they usually measure 5 to 10 cm [6]. The majority of IMs appear as a single mass. If multiple, they are associated with fibrous dysplasia of the bones of the same extremity, known as Mazabraud syndrome [6,7].

The vast majority of patients are asymptomatic, and the myxoma appears as a painless, slowly enlarging, palpable, well-defined, round-shaped mass [4].

The current modes of imaging IMs are ultrasonography, CT and MRI. On ultrasonography, IM appears as a heterogeneous hypoechoic relative to skeletal muscle mass, with well-defined margins. IM usually does not appear capsulated, but sometimes it can have a partial or complete capsule [4]. Before the administration of intravenous contrast, CT reveals a mass of low attenuation (less than that of the muscle), with almost equal appearance of homo- and heterogeneous texture [4]. After the administration of intravenous contrast, CT images reveal an equal percentage of mild enhancement and of absence of enhancement [4]. MRI shows low signal intensity on T1-weighted images and high signal intensity on T2-weighted images, with peripheral or patchy enhancement after injection of gadolinium [4]. CT and MRI may reveal surrounding muscle edema [4].

The treatment of IM is its surgical excision with a wide local excision, and has an excellent prognosis [3]. After the resection of the mass, recurrence can occur in fewer than 5% of cases [3]. Recurrence may be attributable to insufficient resection of the tumor [3].

Recent studies show that the detection of *GNAS1 *mutations has an increased specificity in the diagnosis of IM [8], although testing for *GNAS1 *mutations is not commonly applicable. This makes the diagnosis of IMs difficult before surgical excision.

On histology, IM demonstrates a hypocellular and hypovascular appearance composed of fibroblasts embedded in an abundant myxoid matrix [2]. The abundant myxoid stroma consists of two main macro-molecules: polysaccharide glycosaminoglycans and fibrous structural proteins [9]. However, in some cases of IM, areas of increased cellularity and vascularity can be recognized [5]. This finding does not affect the benign behavior of IM but can mislead clinicians into diagnosing it as myxoid sarcoma [5]. In IM, the mitotic activity is practically absent [6]. On cytopathology, IM usually consists of bland spindle cells [2]. Immunohistology shows expression of vimentin and a myxoid material which is entirely digestible by hyaluronidase [3]. IM shows no reactivity for S-100 protein, unlike myxoid liposarcoma [3].

The differential diagnosis of IM includes myxoid liposarcoma, myxofibrosarcoma, myxoid chondrosarcoma, leiomyosarcoma, embryonal rhabdomyosarcoma, neurofibroma, nerve sheath myxoma or neurothekeoma, synovial sarcoma, aggressive angiomyxoma, dermoid and epidermoid cyst, lipoma, neuroma and ganglioma [1,3,4,6,9].

CA 19.9, also known as sialylated Lewis a-antigen (a blood protein in red blood cells), is an antigen defined by the monoclonal antibody 1116NS 19.9. It was first mentioned by Koprowski *et al*. in 1979 [10]. It is synthesized by normal human pancreatic and biliary ducts and by gastric, colonic, endometrial, salivary and bronchial epithelium. CA 19.9 is considered to be the best serum tumor marker for pancreatobilliary cancer and colorectal cancer. Its reference range is usually 0 to 37 U/mL. CA 19.9 has a 70% to 90% sensitivity and 80% to 90% specificity in detecting pancreatobiliary cancer [10]. Its positive predictive value is 69%, and its negative predictive value is 90% for the detection of pancreatobilliary cancer [10]. False-positive results (31%) have been associated with other pancreatobilliary disorders (for example, gallstones, pancreatitis, cystic fibrosis), inflammatory bowel disease, duodenum ulcer, gastric and colonic polyps, diabetes mellitus, thyroid-related disorders (for example, hypothyroidism), liver disease (for example, hepatitis, alcoholic and non-alcoholic liver disease, polycystic liver disease), splenic cyst, pulmonary problems (for example, pneumonia, bronchogenic cyst, interstitial pulmonary disease), kidney problems (for example, hydronephrosis, renal cyst), collagen vascular disease, female reproductive system disease (for example, endometriosis) and even heavy tea consumption [11].

Our patient was diagnosed with an IM, which was fully resected and had no evidence of recurrence at follow-up six months later. Although it appears to be a typical presentation of IM, the elevated CA 19.9 level, which returned to normal values six months after the resection, was challenging. For this reason, we searched for other possible causes of CA 19.9 elevation, and we submitted our patient to a number of imaging and laboratory studies to rule out other possible diagnoses.

Our patient did not refer to any symptoms related to conditions that elevate CA 19.9, and the commonest types of malignancy that cause this elevation were excluded. Pancreatobilliary and colon malignancies could not be the cause because upper abdominal ultrasonography and colonoscopy results were normal. We also excluded the possibility of benign diseases affecting the liver, pancreas, gallbladder, kidneys, reproductive system, colon and lungs to be the cause of this elevation because upper and lower abdominal ultrasonography, colonoscopy and chest radiography did not reveal any pathology. An increase in CA 19.9 values has also been associated with hypothyroidism [12], but this elevation does not affect euthyroid patients [12]. In our case, our patient was euthyroid before and after the surgical excision, and hypothyroidism was also excluded as a possible reason of CA 19.9 elevation. Taking these into consideration, we assumed that our case could be indicative of an association between CA 19.9 and IM because normal values were restored after the resection. To the best of our knowledge, there has been no previously reported association of serum tumor markers with IM [6].

## Conclusion

IM is a benign soft tissue tumor with an excellent prognosis after its surgical excision. CA 19.9 is a tumor marker associated with malignancies of the pancreatobilliary and colonic tract and with a multitude of benign conditions. Our case raises the question of whether CA 19.9 is also associated with IM and indicates the need for more data to be collected toward this direction.

## Consent

Written informed consent was obtained from the patient for publication of this case report and any accompanying images. A copy of the written consent is available for review by the Editor-in-Chief of this journal.

## Competing interests

The authors declare that they have no competing interests.

## Authors' contributions

DT contributed to conception, writing and critical revision of the manuscript. ESK contributed to research, acquisition of data, analysis, drafting and writing of the manuscript. GID contributed to research, acquisition of the data and writing of the manuscript. PGD contributed to post-operative management, and acquisition and interpretation of the data. AL contributed to post-operative management, writing and critical review of the manuscript. KT assisted in the operation and contributed to post-operative management and manuscript conception. SK carried out the operation and contributed to post-operative management, manuscript conception, acquisition of consent and critical review of the manuscript. All authors read and approved the final manuscript.
